# COVID-19 may have increased global support for universal health coverage: multi-country observational study

**DOI:** 10.3389/fpubh.2023.1213037

**Published:** 2023-08-25

**Authors:** Claudia F. Nisa, Xiaoxi Yan, Bibhas Chakraborty, Pontus Leander, Jocelyn J. Bélanger

**Affiliations:** ^1^Division of Social Sciences, Duke Kunshan University, Kunshan, China; ^2^Global Health Research Center, Duke Kunshan University, Kunshan, China; ^3^New York University Abu Dhabi, Abu Dhabi, United Arab Emirates; ^4^Duke-NUS Medical School, Singapore, Singapore; ^5^College of Liberal Arts and Sciences, Wayne State University, Detroit, MI, United States; ^6^Carnegie-Mellon University Qatar, Qatar Education City, Doha, Qatar

**Keywords:** COVID-19, universal health care, social cohesion, group solidarity, risk assessment, trust

## Abstract

**Introduction:**

The multiple risks generated by the COVID-19 pandemic intensified the debate about healthcare access and coverage. Whether the burden of disease caused by the coronavirus outbreak changed public opinion about healthcare provision remains unclear. In this study, it was specifically examined if the pandemic changed support for governmental intervention in healthcare as a proxy to support for universal health coverage (UHC). It also examined which psychological factors related to the socioeconomic interdependence exposed by the pandemic may be associated with a potential change.

**Methods:**

Online survey data was collected over 18 months (from March 2020 to August 2021) across 73 countries, containing various social attitudes and risk perceptions related to COVID-19. This was a convenience sample composed of voluntary participants (*N* = 3,176; age 18 years and above).

**Results:**

The results show that support for government intervention in healthcare increased across geographical regions, age groups, and gender groups (an average increase of 39%), more than the support for government intervention in other social welfare issues. Factors related to socioeconomic interdependence predicted increased support for government intervention in healthcare, namely, social solidarity (ß = 0.14, *p* < 0.0001), and risk to economic livelihood (ß = 0.09, *p* < 0.0001). Trust in the government to deal with COVID-19 decreased over time, and this negative trajectory predicted a demand for better future government intervention in healthcare (ß = −0.10, *p* = 0.0003).

**Conclusion:**

The COVID-19 pandemic may have been a potential turning point in the global public support for UHC, as evidenced by a higher level of consensus that governments should be guarantors of healthcare.

## Introduction

The massive social and economic disruption generated by the COVID-19 pandemic intensified the debate about healthcare access and coverage (1–3). The COVID-19 pandemic exposed the peril of fragile healthcare systems, where restricted access, low coverage, and high costs aggravated mortality rates and health inequalities at the local and global levels (4, 5).

The goal of this research was to examine the changes in public opinion about healthcare provision, particularly whether governments should be guarantors of healthcare. Assessing the level of agreement about governmental intervention in healthcare is crucial for universal health coverage (UHC) (6–8), an all-embracing target of the 2030 Sustainable Development Goals (9). As defined by the World Health Organization, UHC means that all people have access to the health services they need, when and where they need them, without financial hardship (9). Support for governmental intervention in healthcare is typically aligned with these principles. UHC may come in different forms, but the core idea is that the government steps in with taxpayer money to ensure that every citizen has access to the medical care they need. The common denominator in all paths toward high healthcare access and coverage is always some form of government intervention (10–12); the government is typically expected to play a significant role, leveraging its unique position as regulator, subsidizer, and/or provider.

Some authors claim that public opinion varies regarding whether healthcare provision should fall within the government's scope of action (13). Some others claim that public opinion toward healthcare is not the problem, but the cost (14), and that people are unaware of the trade-offs between competing social objectives and limited public finances. Nevertheless, favorable public opinion for government intervention is politically relevant to push forward health policies that may require substantial public investment, higher social security contributions, or higher health insurance premiums. Establishing whether COVID-19 may have created a window of opportunity for governments to act about healthcare is an important empirical point to make. Although COVID-19 heightened the conceptual and policy debate about healthcare coverage, to this date, limited empirical accounts have been reported about public opinion changes prompted by the pandemic and their relation to downstream healthcare preferences. This study aims to address this gap.

Using the observational data from an 18-month survey in 73 countries covering the critical period of the COVID-19 pandemic (March 2020 to August 2021) (15), this study examines (a) the self-reported change in support for government intervention in healthcare and (b) the psychological factors that predicted this change. The psychological factors selected for analysis as potential drivers of change were informed by past research and policy discussions, namely, (1) social solidarity, (2) risk perceptions, and (3) trust in government vs. private business to provide healthcare.

Jointly, these predictors appraise different facets of socioeconomic interdependence (16), which was made salient in the prolonged pandemic shared experience. Essentially, socioeconomic interdependence is a core justification for UHC and the welfare state. Advocating for health as a fundamental human right is grounded in social solidarity and conceptualized as an individual entitlement to health benefits regardless of one's ability to pay (17). Social solidarity conveys the principle of communal help between the members of social groups (e.g., countries) to achieve social wellbeing (18). A social solidarity standpoint toward healthcare embraces the principles of equality and equity and tends to hold—and trust—the government to be accountable for the provision of healthcare. Trust in government has been shown to correlate with trust in health organizations and demand for healthcare services (19, 20). However, government (perceived) failures in terms of speed or efficiency of the services provided to the population may lead to shifting—or at least shared—preferences for private businesses to be healthcare providers (21).

Furthermore, unpredictable and catastrophic risk is also central to the welfare state and UHC (22), which advocates for reciprocal aid when facing threats. Risk-sharing is often mediated by the government in the form of income redistribution and social security, from the wealthy to the poor or the healthy to the sick (23). The COVID-19 pandemic has elicited strong (shared) perceptions of risk, both about health and the economy, and these risk perceptions have been shown to influence a variety of social attitudes (24). Previous research has shown that experimentally manipulating threats to healthcare increased political liberalism (25) and that experiencing the loss of employment/health insurance was associated with support for UHC (26, 27).

However, thus far, there is no research linking these factors to public opinion about healthcare provision in the prolonged context of the COVID-19 pandemic. This study shows that support for government intervention in healthcare increased across geographical regions, age groups, and gender groups, more than support for government intervention in old age and unemployment. Perceptions of social solidarity and risk to economic livelihood increased support for government intervention in healthcare, whereas trust in the government showed a negative association—interpreted as a demand for better future government intervention in healthcare. Taken together, the results suggest that the COVID-19 pandemic may have been a potential turning point in global public support for UHC, as evidenced by a higher level of consensus that governments should be guarantors of healthcare. The universal healthcare agenda may have a higher likelihood to be accepted during or in the aftermath of infectious outbreaks, economic crises, or natural disasters—as these events are likely to promote negative financial instability while also buffering bonds of social solidarity.

## Methods

### Study design and sample

Data for this observational study were obtained from the global Psycorona project (15), which focused on how people feel and think about the coronavirus epidemic. The questions asked to participants covered a large variety of topics, from emotional states, social attitudes, and healthcare behaviors to policy support for different COVID-19 measures (15). All waves, data, and codebook are publicly available at the project website (15), with most items being adapted from previously validated psychometric scales. Volunteer participants from a convenience sample completed a baseline cross-sectional survey (in March 2020) distributed via word-of-mouth, personal social networks, and platforms such as Facebook with paid ads. These data were not available via the Ministries of Health. A subset of participants signed up for a longitudinal study involving follow-up surveys over the course of the pandemic (until August 2021). This study focused on a cohort of participants who completed at least two waves between March and December 2020 (waves 0–16) measuring predictors and at least one wave measuring outcomes: wave 18 (February 2021) and wave 22 (August 2021) (*N* = 3,176). The survey was translated into 30 languages and distributed by members of the research team (consisting of over 100 psychologists) in their respective countries using social media campaigns, press releases, and social and academic networks. Personal identifiers were removed from all sections of the manuscript, including Supplementary material and the public dataset.

The countries selected for analysis were determined by the nationality of the co-authors of the Psycorona project; each researcher/team was responsible for collecting data in his/her own country or where personal networks allowed for online data collection. Data were reported from 73 countries: Algeria; Argentina; Australia; Austria; Bangladesh; Bosnia and Herzegovina; Brazil; Belgium; Bulgaria; Cambodia; Canada; Chile; China; Colombia; Costa Rica; Croatia; Cyprus; Czech Republic; Ecuador; Egypt; El Salvador; Estonia; Finland; France; Georgia; Germany; Greece; Hungary; Hong Kong; India; Indonesia; Iran; Ireland; Israel; Italy; Iraq; Japan; Jordan; Kazakhstan; Kosovo; Lebanon; Lithuania; Luxembourg; Malaysia; Mexico; the Netherlands; New Zealand; Panama; Peru; Philippines; Poland; Russia; Romania; Saudi Arabia; Serbia; Singapore; Slovakia; South Africa; South Korea; Spain; Sweden; Switzerland; Taiwan; Thailand; Trinidad and Tobago; Turkey; Ukraine; UAE; UK; USA; Uruguay; Venezuela; and Vietnam.

The sample (*N* = 3,176) was gender unbalanced (67% women), with 38% up to 44 years of age and 62% aged above 45 years (range 18–85 years). Less than half of the participants were educated up to higher education (43%), and the remaining had completed higher education (26% with an undergraduate degree and 30% with postgraduate studies).

Data quality control was conducted by examining IP addresses to detect potential duplicate responders and removing participants from the database whose answers indicated random responses. These countries covered various levels of economic development as well as different temporal stages of the COVID-19 pandemic, suggesting the need for the country-level covariates presented below.

### Predictors and covariates

The predictor variables selected for the current analysis were deemed more conceptually relevant for the topic under research and were taken from the databank of the Psycorona project. Indicators of perceived socioeconomic interdependence (i.e., social solidarity, risk perceptions, and trust in government and private business) were measured in 16 waves from March to December 2020. Predictors were measured at multiple points from March 2020 to December 2020 (waves 0–16). The factors were measured as follows: (a) Social solidarity: “I feel a sense of solidarity with people in my country” (from −3 = Strongly disagree to 3 = Strongly agree); (b) Risk perception about the economy: “How likely is it that your personal situation will get worse due to economic consequences of coronavirus” (from 1 = Exceptionally unlikely to 8 = Already happened); (c) Risk health perception: “How likely is you will get infected with coronavirus” [SIC](from 1 = Exceptionally unlikely to 8 = Already happened); (d) Trust in government: “To what extent do you trust the government in your country?” (from 1 = Not at all to 5 = Very much); and (e) Trust in business: “To what extent do you trust the private businesses in your country?” (from 1 = Not at all to 5 = Very much).

Several individual and country-level predictors were added as covariates in multilevel regression models. Individual-level covariates were sociodemographic variables (age, gender, and education). Country-level covariates were not present in the Psycorona databank, which focused only on individual-level psychological variables. Conceptually relevant country-level covariates were selected and matched in the database to each participant according to their country of origin. These variables included (1) unemployment rate (as % of the labor force—from World Bank 2020 data^1^), (2) general health expenditure (as % GDP—from World Bank 2020 data), (3) out-of-pocket health payments (as % total health expenditure—from World Bank 2020 data), and (4) case-fatality rates (# deaths from COVID-19/ # positive COVID-19 cases—from World in Data, retrieved June 2022).

### Outcome measures

The dependent variables were specifically designed and included in the Psycorona survey to address the research question examined in this study. The primary outcome (changes in support for government intervention in healthcare) was measured in February 2021 and August 2021. Secondary outcomes included asking participants whether the pandemic had changed their views about the government providing social protection in old age and unemployment. The questions related to these outcomes were asked in tandem: “Has the pandemic changed your views on these topics? a) The government should provide a decent standard of living for the old; b) The government should provide a decent standard of living for the unemployed; c) The government should provide healthcare for the sick” (from −3 = disagree much more now to 3 = agree much more now). These items were examined individually and were informative in their own right. However, these three items combined can be interpreted as an overall measure of political attitudes (revealing a good internal consistency, Cronbach's α = 0.880) and allowed to maintain tacitly the specific goal of the study, i.e., views about healthcare.

### Statistical analysis

To the best of our knowledge, previous literature was scarce to confidently propose or guide hypotheses about the effect of a global pandemic on future preferences about healthcare provision. Thus, there was no formalization nor pre-registration of hypotheses.

Descriptive statistics comparing average support for government intervention in February 2021 vs. August 2021, as well as for different types of government intervention (unemployment, old age, and healthcare), were conducted using paired *t*-tests. Differences between geographical regions in support of government intervention were tested using ANOVA and Bonferroni *post-hoc* tests.

Using multiple regression analyses, it was examined to what extent the conceptually selected factors (social solidarity, economic risk, health risk, and trust in government) predicted support for government intervention in healthcare. These multiple linear regression models included both unadjusted and adjusted estimates (controlling for the covariates described above at the individual and country levels, theoretically justified). Details about data distribution are presented in Supplementary material.

The initial multiple regression models did not account for the multiple country origins of the participants. Therefore, sensitivity analyses applied multilevel, hierarchical models to understand the effects of controlling for person-level predictors, considering the random variations across nations. The predictors at the individual level were group mean-centering by country (and scaling is done by dividing the (centered) columns of x by their standard deviations). Country-level variables used grand mean centering, given that these have a single value for each country. These multilevel models were implemented using R and the package lme4. The intraclass correlation coefficient (ICC) was estimated to describe the correlation among observations within the countries. The ICC is also equivalent to the variance partition coefficient, which can be interpreted as the proportion of variation that is due to variation between countries. Extreme outliers (more than 3SD from the mean) were excluded, and all reported *p-*values are two-sided.

## Results

### Descriptive analysis

**Figure 1:**
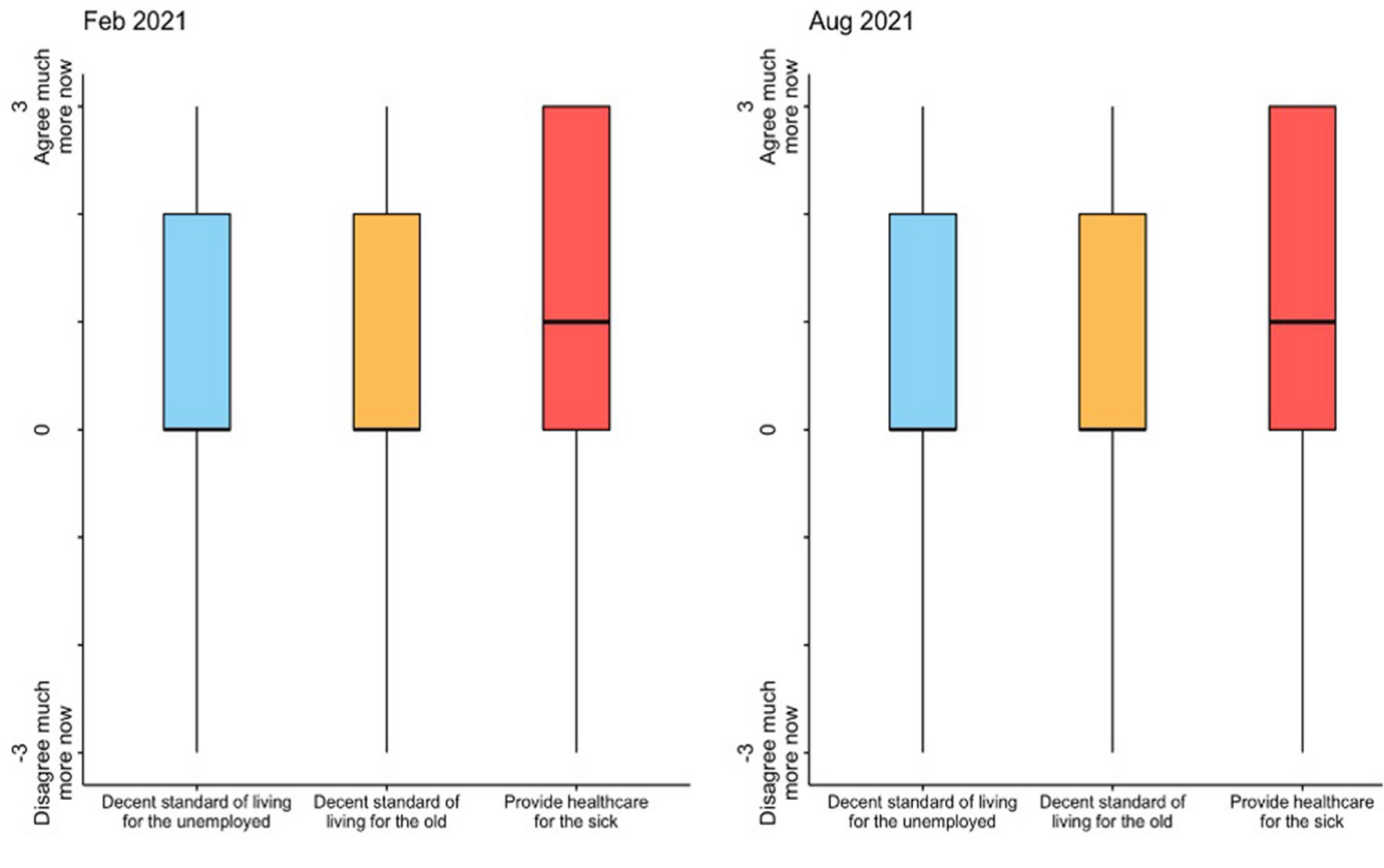
Average change in support for government intervention in social welfare in February 2021 **(left)** and August 2021 **(right)**. Questions: Has the pandemic changed your views on these topics? (a) The government should provide a decent standard of living for the old; (b) The government should provide a decent standard of living for the unemployed; (c) The government should provide healthcare for the sick” (from −3 = disagree much more now to 3 = agree much more now).

The main outcomes are shown in Figure 1. Participants perceived their support for government intervention in healthcare had increased due to the pandemic (mean = 1.16, SD = 1.35; equivalent to 38.6% average increase, 95% CI 36.6%, 40.6%), above the increase in support for government intervention to protect the older adults (mean = 0.99, SD = 1.33; paired *t*-test diff *p* < 0.001) and the unemployed (mean = 0.88, SD = 1.28; paired *t*-test diff *p* < 0.001) (results for February 2021, *N* = 3,169). This change was stable after 6 months; there was no within-subject average difference between February and August 2021 regarding support for government intervention in healthcare (paired *t*-test *p* = 0.302).

Higher support for government intervention in healthcare due to the COVID-19 pandemic is consistent across gender and age groups. Both women and men equally support more government intervention in healthcare (mean diff = −0.039, *p* = 0.969), over other forms of governmental intervention in social welfare (all paired *t*-test *p* < 0.05). Similarly, all age groups reported higher support for government intervention in healthcare than in other areas (all paired *t*-tests *p* < 0.01 comparing different support for social welfare areas with each age group).

**Figure 2:**
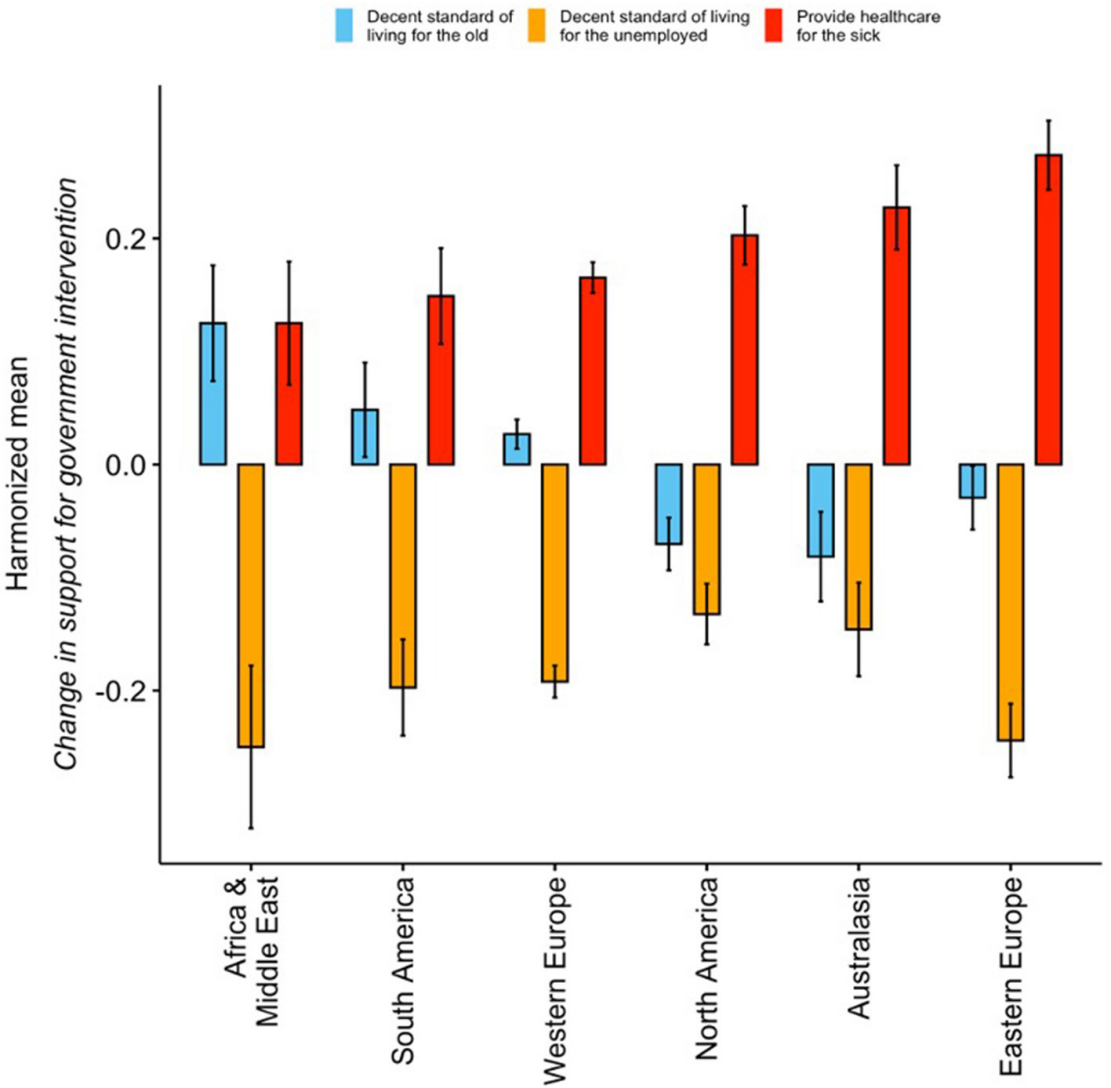
Change in support for government intervention due to COVID-19 per welfare issue, per region (August 2021) – Harmonized means. North America: Canada; USA; South America: Venezuela; Peru; Mexico; El Salvador; Colombia; Chile; Brazil; Argentina; Trinidad and Tobago; Panama; Ecuador; Costa Rica; Uruguay; Western Europe: UK; Spain; the Netherlands; Italy; Greece; Germany; France; Belgium; Sweden; Switzerland; Luxembourg; Ireland; Finland; Cyprus; Austria; Eastern Europe: Ukraine; Turkey; Russia; Romania; Serbia; Poland; Lithuania; Kosovo; Kazakhstan; Hungary; Estonia; Czech Republic; Croatia; Bulgaria; Bosnia and Herzegovina; Slovakia; Georgia; Africa & Middle East: UAE; South Africa; Saudi Arabia; Lebanon; Jordan; Israel; Iran; Algeria; Iraq; Egypt; Australasia: Vietnam; Thailand; Singapore; Philippines; Malaysia; Japan; Indonesia; India; Bangladesh; Australia; Taiwan; South Korea; New Zealand; Hong Kong; China; Cambodia.

Harmonized^2^ mean differences per geographical region are shown in Figure 2. The results show that, across the world, participants perceived their support for government intervention in healthcare to have increased more than the support for government intervention in other welfare areas (all paired *t*-test *p* < 0.01), except for Africa and the Middle East, where support for government caring for the older adults increased as much as for healthcare (paired *t*-test *p* = 0.678). Between-region differences in increased support for government intervention in healthcare were only identified between Eastern and Western Europe (Bonferroni *post-hoc p* = 0.006), with Eastern Europe reporting the largest increase.

### Factors predicting change in support for government intervention in healthcare

Multiple linear regression analyses averaging each predictor from the baseline (wave 0) to wave 16 (Table 1) show that both economic risk perception and feelings of social solidarity due to COVID-19 were positive predictors of increased support for government intervention in health. Economic risk perception showed a consistent association with this outcome in both February 2021 (unadjusted ß = 0.14, *p* < 0.0001; adjusted ß = 0.09, *p* < 0.0001) and August 2021 (unadjusted ß = 0.14, *p* < 0.0001; adjusted ß = 0.09, *p* < 0.0001). Similarly, the association of social solidarity with support for government intervention in healthcare was significant in February 2021 (unadjusted ß = 0.13, *p* < 0.0001; adjusted ß = 0.10, *p* < 0.0001) and August 2021 (unadjusted ß = 0.17, *p* < 0.0001; adjusted ß = 0.14, *p* < 0.0001).

**Table 1:** Multiple linear regression averaging waves w0–w16 (March–December 2020) predicting change in support for government intervention in healthcare (measuring in February 2021 and August 2021).

**Predictors 2020**	**February 2021**		**August 2021**	
	**Unadjusted (1)**	**Adjusted (2)**	**Unadjusted (3)**	**Adjusted (4)**
Constant	0.94^***^ (0.65, 1.23)	1.13^***^ (0.62, 1.64)	1.04^***^ (0.74, 1.34)	1.14^***^ (0.61, 1.68)
Health risk	−0.01 (−0.06, 0.04)	0.004 (−0.04, 0.05)	−0.05^*^ (−0.10, −0.001)	−0.02 (−0.07, 0.03)
Economic risk	0.14^***^ (0.11, 0.18)	0.09^***^ (0.05, 0.12)	0.14^***^ (0.11, 0.18)	0.09^***^ (0.05, 0.13)
Trust government	−0.08^**^ (−0.13, −0.03)	−0.08^**^ (−0.14, −0.03)	−0.10^***^ (−0.15, −0.04)	−0.10^***^ (−0.16, −0.05)
Trust business	−0.06^*^ (−0.13, −0.002)	−0.04 (−0.10, 0.02)	−0.04 (−0.11, 0.02)	−0.02 (−0.08, 0.04)
Social solidarity	0.13^***^ (0.09, 0.17)	0.10^***^ (0.06, 0.14)	0.17^***^ (0.12, 0.21)	0.14^***^ (0.09, 0.18)
Age		−0.005 (−0.04, 0.03)		0.01 (−0.02, 0.05)
Gender		−0.03 (−0.13, 0.08)		0.01 (−0.10, 0.11)
Education		−0.04^*^ (−0.08, −0.01)		−0.07^***^ (−0.11, −0.04)
Unemployment rate		0.03^***^ (0.02, 0.04)		0.02^***^ (0.01, 0.03)
Health expenditure		−0.02 (−0.04, 0.001)		−0.01 (−0.03, 0.001)
Out-of-pocket pay		0.01^***^ (0.005, 0.02)		0.01^***^ (0.01, 0.02)
Case fatality		−1.27 (−2.71, 0.16)		−2.01^*^ (−3.48, −0.53)
Observations	2,968	2,968	2,602	2,602
R^2^	0.04	0.07	0.05	0.08
Adjusted R^2^	0.04	0.07	0.04	0.07
Residual Std. Error	1.35 (df = 2,962)	1.33 (df = 2,955)	1.31 (df = 2,596)	1.29 (df = 2,589)
F-statistic	25.55^***^	18.28^***^	25.21^***^	18.14^***^

* *p* < 0.05;

** *p* < 0.01;

*** *p* < 0.001. Unadjusted models examining only individual-level variables. Adjusted models include estimates for age, gender, and educational level (individual-level covariates) and unemployment rate, %GDP expenditure in healthcare, out-of-pocket payments, and COVID-19 case-fatality rate.

Both health risk perception and trust in business show no significant association with the outcome, suggesting that the perceived likelihood of getting infected with the virus and placing trust in the private sector to deal with COVID-19 played no role in changing individuals' preferences for healthcare provision.

An interesting negative association was consistently found between trust in government and support for government intervention in healthcare: the lower the reported trust in government, the more participants agreed that government should provide healthcare for the sick (unadjusted ß = −0.08, *p* = 0.004; adjusted ß = −0.08, *p* = 0.002 February 2021; unadjusted ß = −0.10, *p* = 0.001; adjusted ß = −0.10, *p* = 0.0003 August 2021). However, attitudes toward pro-government intervention typically tend to be positively related to trust in the government. This negative association is interpreted as a demand for better future government intervention in healthcare; the less people trust the government to cope with COVID-19, the more they think that the government should be doing a better job in the future providing healthcare for the sick.

The results for individual and country-level covariates in Table 1 are detailed and discussed in Supplementary material.

### Sensitivity analyses

Sensitivity analyses were conducted to determine the robustness of these results, including restricting the analysis to countries with *N* > 100 (Table 2), comparing low- and middle-income countries vs. high-income countries (Supplementary Table 3), and conducting multilevel modeling analysis (Supplementary material). With respect to restricting the analysis to countries with N > 100, this confined the analysis to the United States and European countries. Thus, separate comparative models were conducted using the data from August 2021 (Table 2).

**Table 2:** Multiple linear regression averaging waves w0–w16 (March–December 2020) predicting change in support for government intervention in healthcare (measuring in August 2021).

**Predictors 2020**	**USA**		**Europe**	
	**Unadjusted (1)**	**Adjusted (2)**	**Unadjusted (3)**	**Adjusted (4)**
Constant	1.22^**^ (0.44, 2.00)	1.65^**^ (0.51, 2.79)	0.64^**^ (0.20, 1.08)	−1.36 (−3.87, 1.16)
Health risk	0.03 (−0.09, 0.16)	0.05 (−0.08, 0.17)	−0.01 (−0.09, 0.06)	0.01 (−0.06, 0.09)
Economic risk	0.09 (−0.001, 0.17)	0.08 (−0.01, 0.17)	0.16^***^ (0.11, 0.21)	0.10^***^ (0.04, 0.15)
Trust government	−0.25^*^ (−0.45, −0.04)	−0.27^*^ (−0.48, −0.07)	−0.06 (−0.14, 0.01)	−0.04 (−0.12, 0.04)
Trust business	−0.08 (−0.27, 0.12)	−0.06 (−0.25, 0.14)	−0.06 (−0.15, 0.04)	−0.05 (−0.14, 0.05)
Social solidarity	0.12^*^ (0.01, 0.23)	0.12^*^ (0.005, 0.23)	0.22^***^ (0.15, 0.29)	0.17^***^ (0.09, 0.24)
Age		0.000 (−0.00, 0.09)		0.04 (−0.01, 0.09)
Gender		−0.0003 (−0.30, 0.30)		−0.04 (−0.19, 0.12)
Education		−0.09 (−0.19, 0.01)		−0.09^***^ (−0.14, −0.04)
Unemployment rate				0.03 (−0.0002, 0.06)
Health expenditure				0.14 (−0.04, 0.32)
Out-of-pocket pay				0.04^***^ (0.01, 0.06)
Case fatality				0.61 (−2.79, 4.02)
Observations	417	417	1,160	1,160
R^2^	0.04	0.05	0.07	0.12
Adjusted R^2^	0.03	0.03	0.07	0.11
Residual Std. Error	1.36 (df = 411)	1.36 (df = 408)	1.27 (df = 1,154)	1.24 (df = 1,147)
F-statistic	3.33^**^	2.45^*^	17.53^***^	13.26^***^

* *p* < 0.05;

** *p* < 0.01;

*** *p* < 0.001. Unadjusted models examining only individual-level variables. Adjusted models include estimates for age, gender, and educational level (individual-level covariates) and unemployment rate, %GDP expenditure in healthcare, out-of-pocket payments, and COVID-19 case-fatality rate. Countries included Europe: Germany; Greece; the Netherlands; Spain; and the United Kingdom. Only Europe has variations in country-level covariates, thus no such information is shown for the United States.

Economic risk perception was only associated with support for government intervention in healthcare in Europe (unadjusted ß = 0.16, *p* < 0.0001; adjusted ß = 0.10, *p* = 0.0005). Specific to the United States was the negative and moderate association between trust in government and support for government intervention in healthcare (unadjusted ß = 0.25, *p* = 0.02; adjusted ß = 0.27, *p* = 0.02). Common to both sides of the Atlantic was the positive association between social solidarity and support for government intervention (unadjusted ß = 0.22, *p* < 0.0001; adjusted ß = 0.17, *p* = 0.0001 vs. US unadjusted ß = 0.12, *p* = 0.04; adjusted ß = 0.12, *p* = 0.05). The results for individual and country-level covariates in Table 2 are also detailed and discussed in Supplementary material.

The comparison between low- and middle-income countries vs. high-income countries is presented in Supplementary Table 3. Low- and middle-income countries showed the only instance where perceived health risk was associated with support for government intervention, yet this was a negative association (unadjusted ß = −0.11 *p* = 0.05; adjusted ß = −0.11, *p* = 0.05). The more people in low- and middle-income countries perceive a high likelihood of contracting the coronavirus, the less they support government intervention in healthcare. This may suggest perceived government inefficiency or corruption. Consistent between low- and middle-income countries and high-income countries is the effect of economic risk perception, social solidarity (both positive associations), and trust in government (a negative association). Multilevel models largely corroborate the results from multiple linear regression models (Supplementary Table 4).

## Discussion

This study presents empirical evidence that the COVID-19 pandemic may have been a potential turning point in global public support for UHC, as evidenced by a higher level of consensus that governments should be guarantors of healthcare. Globally, individuals perceived an increase in their support for government intervention in healthcare due to COVID-19 across geographical regions, genders, and age groups. This increase was significantly higher than support for the government caring for older adults and the unemployed—as comparable important social welfare issues were also called into question by the COVID-19 pandemic. This result may reflect that people perceived that governments were better able to manage the collateral unemployment and financial instability than they could manage the health burden resulting from the coronavirus, thus demanding better future governmental action in the healthcare domain.

The most consistent factor that predicted the increase in support for government intervention in healthcare was social solidarity. This positive association was found globally, across countries with different political systems and economic development. It has been proposed that social solidarity was the bonding force that helped people deal with social distance during the persistent lockdowns (16, 17, 28). Collectively experiencing negative situations has been shown to motivate people to help each other and foster a willingness to engage in prosocial behavior (29). Faced with the prolonged health burden caused by the coronavirus, people may support more and better government interventions in healthcare as a means to guarantee affordable access and health coverage and to mitigate the suffering experienced or anticipated by family, friends, and other fellow human beings.

The second-most consistent factor that predicted the increase in support for government intervention in healthcare was the economic risk caused by COVID-19. The economic risk created by the pandemic and its implications for social attitudes and behaviors have been largely underestimated and underexamined; most studies have focused on perceptions of risk concerning getting infected. There is research showing that perceived economic risk—and not health risk—was the main predictor of a variety of mitigation behaviors and policy support for COVID-19 containment (24). The more people perceived a personal risk of suffering economic losses due to the pandemic, the more they frequently wash their hands, avoid crowds, socially isolate, support mandatory vaccination, accept mandatory quarantine when diagnosed with coronavirus or when exposed to the virus, and support reporting suspected COVID-19 cases.

Taken together, these results suggest that the universal healthcare agenda may have a higher likelihood to be accepted during or in the aftermath of infectious outbreaks, economic crises, or natural disasters—as events likely to promote negative economic instability while also buffering bonds of social solidarity. Such contexts appear to open receptiveness to the concept of health as a human right to be safeguarded, possibly increasing the acceptance of the costs that may be associated with (universal) public healthcare (e.g., higher taxes and insurance premiums).

Trust in the government was also a significant predictor in several models, but shows a peculiar, consistent, negative association with support for government intervention. Considering past research (e.g., 19–21), this negative association seems counterintuitive. These results suggest that the pandemic (and perhaps also other acute infectious diseases, crises, or natural disasters) has drawn attention to the vital need for affordable access to healthcare and uncovered how much people actually expect the government to intervene to ensure healthcare when needed—despite not necessarily trusting the government apparatus. Furthermore, the negative perceptions about the (in)ability of governmental action to stop COVID-19 over time appear to have ignited a greater demand for better governmental intervention in the future.

With less to no association with the increased support for government intervention in healthcare were perceived health risks and trust in private businesses. Although the private sector is considered an important partner in healthcare provision (30, 31), trust in businesses has not been a particularly discussed topic during the pandemic, with businesses often portrayed as victims of the lockdowns and social distancing. Hence, this may help us understand why this specific type of social trust has no role in healthcare preferences. However, the absence of association with health risk is more remarkable. Perceived vulnerabilities about one's own health due to COVID-19 do not appear to be reliable factors in predicting preferences for healthcare provision, at least when other important variables are controlled for. Worldwide, perceived health risk has been consistently associated with emotional distress and mental health challenges (32–35), but these factors appear unrelated to preferences about healthcare provision.

This study has some limitations that warrant discussion. Although the total sample size is large for a longitudinal study spanning 18 months (*N* = 3,176), taking a country-level approach was limited because our total sample size is smaller than other papers published on cross-country comparisons regarding psychological or behavioral implications from the COVID-19 pandemic (e.g., 24, 29). However, multi-country data collection was sustained over 18 months, covering crucial periods of the pandemic, from the initial stages to more return-to-normal periods, from pre-to-post vaccine development and rollout. This is a much rarer contribution to the literature. Many previous publications have reported much larger samples, but from a single point in time, or from two or three time points. Moreover, our country samples are not nationally representative samples, but convenience and snowball samples. Although the choice of countries was not a deliberate choice but a choice of convenience based on the Psycorona co-authors, data nonetheless were collected on all continents and in a variety of countries with very distinct socioeconomic and political characteristics, including a large array of low- and middle-income countries. The generalizability of results is unclear given that samples were not randomly selected and nationally representative, but survey participants exemplified the active populations 18–65 years old from dozens of countries, in terms of voting age and covering a range of educational levels—an appropriate target population for the research questions under analysis.

Another important aspect to discuss is that our outcomes were not measured as before-and-after differences. Ideally, support for government intervention in healthcare (as well as in old age and unemployment) should have been asked earlier in 2020 or have some pre-pandemic comparative assessment of these factors. Despite this limitation—the assessment of the outcomes only in 2021—two data points in 2021 were collected to guarantee reliability and sensitivity analyses. Moreover, it was established that people can introspect to what extent the pandemic has changed their views on different topics, distinguishing between topics about which their attitudes have changed (more or less) or not changed at all. Notwithstanding the inability to compare how people with different pre-pandemic political attitudes changed over time in their views about government intervention in healthcare, the focus of the study was on the average perceived change—regardless of the participants' baseline point. Thus, these results can be interpreted as a psychological (if not real) average shift toward more liberal political attitudes due to the pandemic. This is characterized by a more positive attitude toward government intervention in social welfare promotion.

Whether this tendency will be sustained over time remains to be established. Catastrophic situations may increase the salience of how much government support is expected and demanded to deal with unpredictable hazards, and this salience may be temporary or fundamentally shift social attitudes in a sustained way.

## Conclusion

The UHC agenda may have a higher likelihood to be accepted during or in the aftermath of infectious outbreaks, economic crises, or natural disasters—as these events are likely to promote negative financial instability while also buffering bonds of social solidarity. Pandemics may open receptiveness to the concept of health as a human right to be safeguarded, possibly increasing the acceptance of the costs that may be associated with UHC.

## Data availability statement

The datasets presented in this study can be found in online repositories. The names of the repository/repositories and accession number(s) can be found at: https://doi.org/10.6084/m9.figshare.23994306.

## Ethics statement

The studies involving humans were approved by the Institutional Review Board at New York University Abu Dhabi (protocol HRPP-2020-42) and the Ethics Committee of Psychology at Groningen University (protocol PSY-1920-S-0390). The studies were conducted in accordance with the local legislation and institutional requirements. The participants provided their written informed consent to participate in this study.

## Author contributions

CN was involved in all stages of the research. XY and BC made substantial contributions to data analysis and result interpretation. PL and JB made substantial contributions to drafting the article and revised it multiple times critically for important intellectual content. All authors were involved in the final approval of the version to be published.

## Footnotes

^1^ https://data.worldbank.org/

^2^Harmonization is required when comparing survey answers from different countries, under the assumption that there may be cultural patterns in the way people interpret rating scales. Harmonization was achieved by creating the mean of all three outcome items and subtracting this mean for each individual item. These adjusted items were then aggregated per region according to the groups presented below Figure 2.
