# Supplementary material for: COVID-19 may have increased global support for universal health coverage: multi-country observational study
Source: Front Public Health. 2023 Aug 25;11:1213037. doi: 10.3389/fpubh.2023.1213037 (PMC10486985; doi:10.3389/fpubh.2023.1213037)
Supplement: Supplementary file 1 [file Table_1.DOCX]

Supplementary Material

COVID-19 may have increased global support for universal health coverage: Multicountry observational study

**Claudia Nisa*, Xiaoxi Yan, Bibhas Chakraborty, Pontus Leander, Jocelyn Belanger**

*** Correspondence:** Corresponding Author: [cfn6@duke.edu](mailto:cfn6@duke.edu)

# Supplementary Tables

**
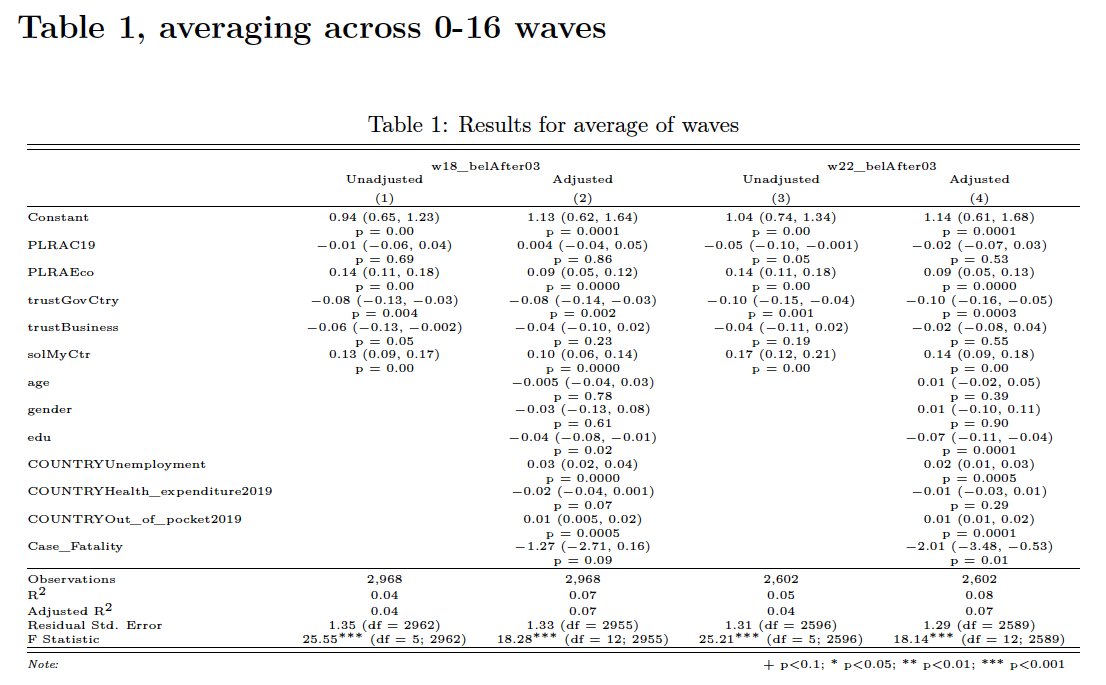
**

**
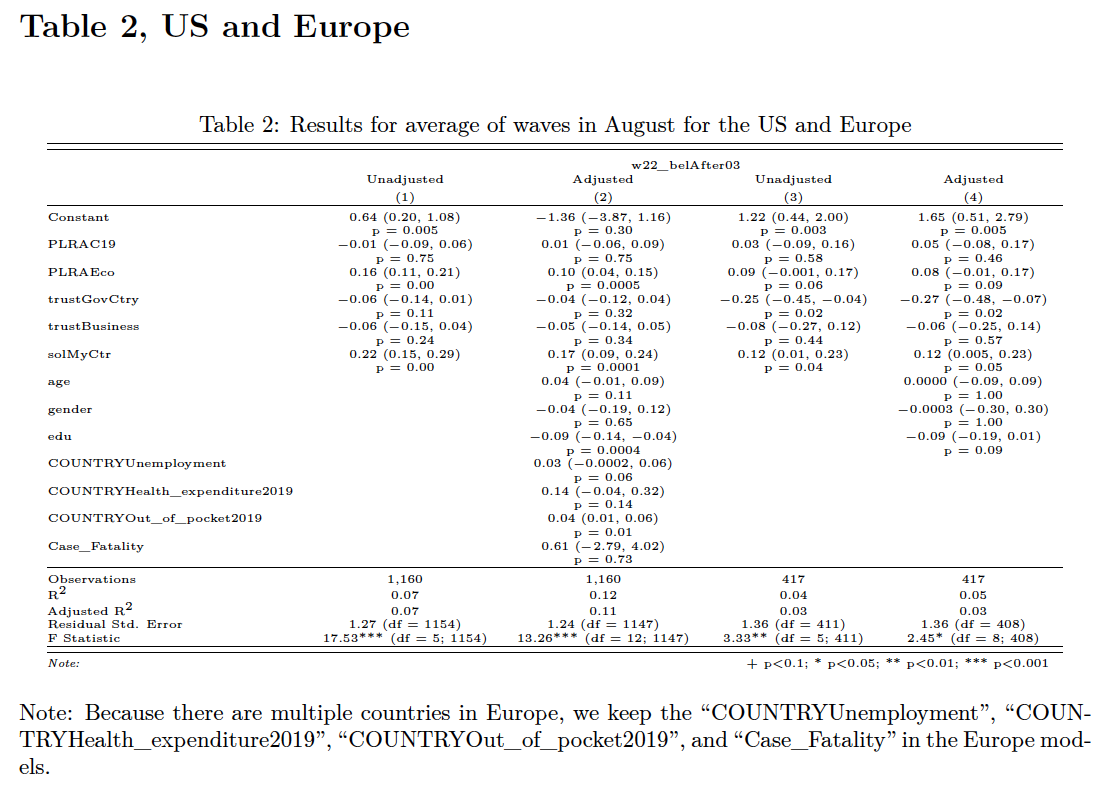
**

**
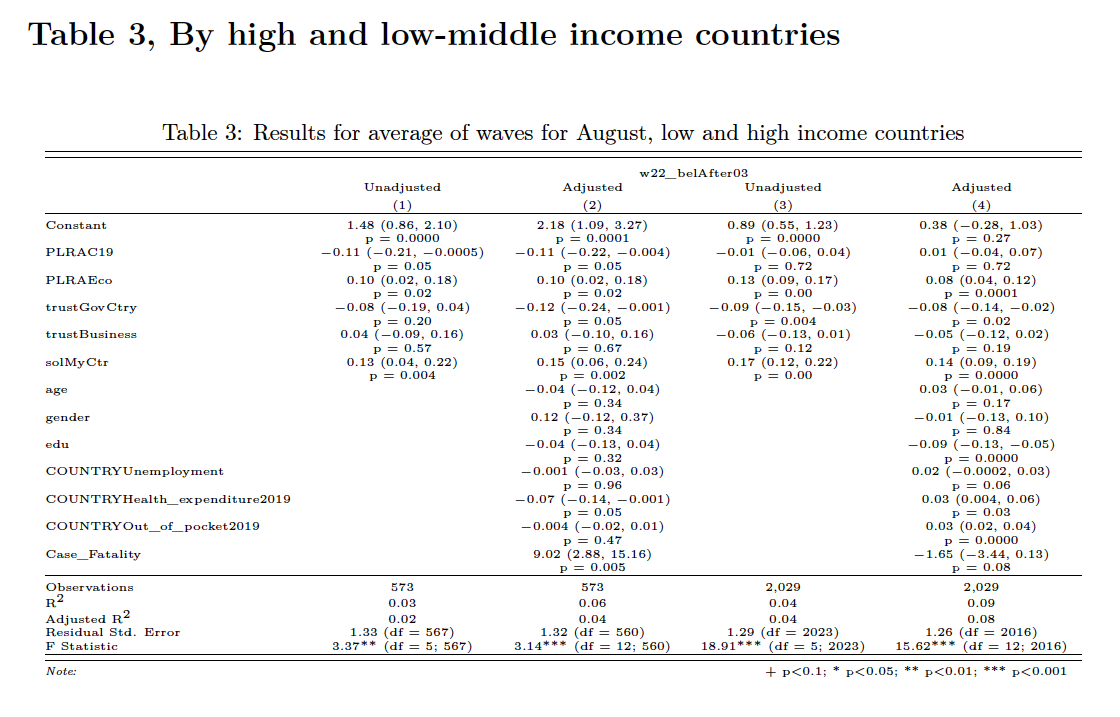
**

**
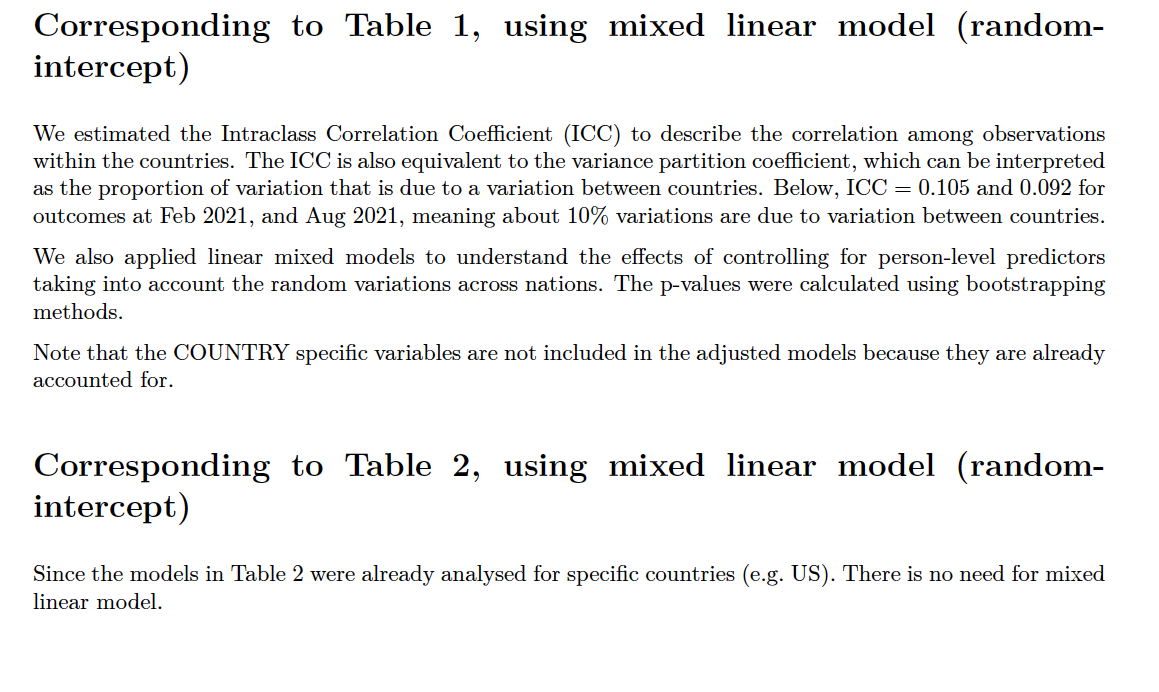
**

**
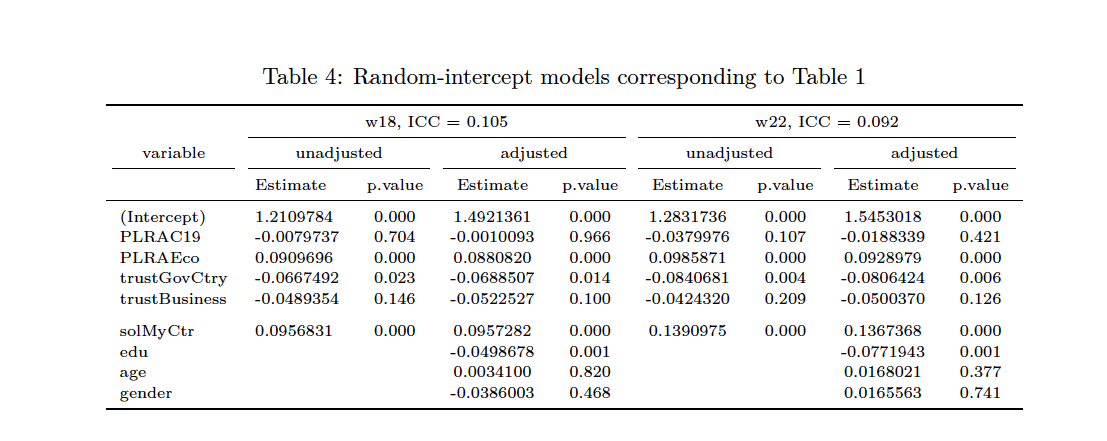
**

**
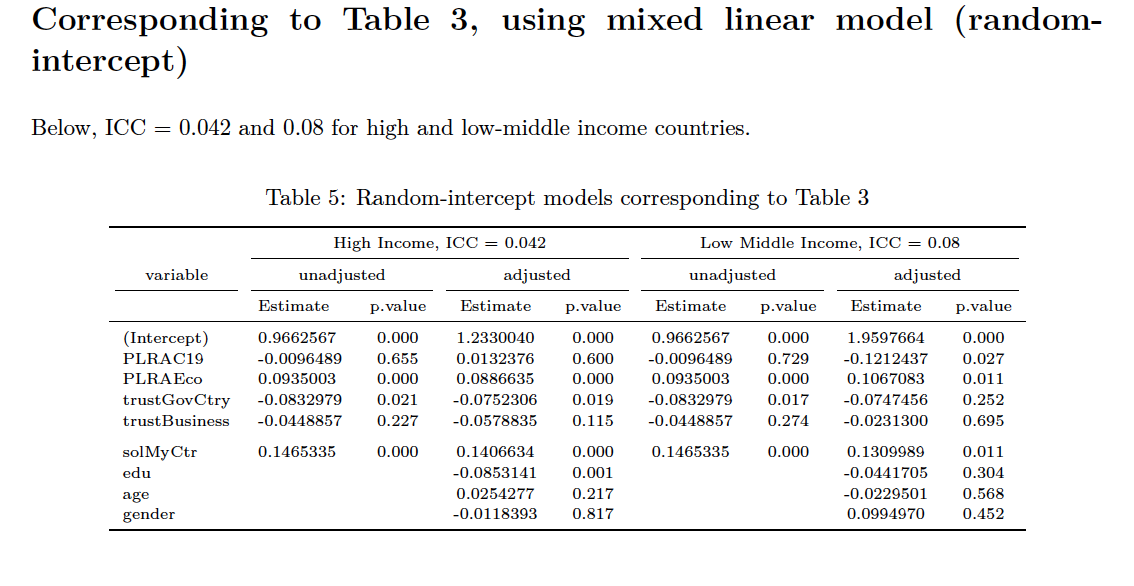
**

# Supplementary Results

Regarding Supplementary Table 1, sociodemographic variables show no significant association with support for government intervention, except educational level (β=-.04 p=.02 February 2021; β=-.07 p=.0001 August 2021). Less educated people tend to support more the government intervening in healthcare, even controlling for perceptions of economic risk, which could account for income vulnerabilities among the less educated. Regarding country-level covariates, adjusted for in Models 2 and 4, results showed consistently (small), significant associations with unemployment rates and out-of-pockets health expenses at both time points. Thus, higher unemployment rates (β=.03 p<.0001 February 2021; β=.02 p=.0005 August 2021) and higher direct household payments to health services (β=.01 p=.0005 February 2021; β=.01 p=.0001 August 2021) are both associated with greater support for government intervention in healthcare.

Lastly, there was a strong negative association between the COVID-19 case-fatality ratio and support for government intervention in healthcare (β=-2.01 p=.01 August 2021). A lower ratio means more deaths per total positive COVID-19 cases, so this negative association suggests that the driving factor behind support for government intervention globally is an exponential number of positive cases, which reduces the case-fatality ratio, given that the mortality rate per 100,000 people tends to be relatively stable (*30*).

Regarding Supplementary Table 2, the negative association with educational level (β=-.09 p=.0004) was also only identified in Europe. Country-level differences within European countries also point to a positive relationship with out-of-pocket health payments (β=.04 p=.01).

In Supplementary Table 3, there is a negative association between the percentage of public investment in healthcare, and support for government intervention in healthcare (β=-.07 p=.05). The factor with an extremely high association with support for government intervention in healthcare in low-and-middle income countries is case-fatality rates (β=9.02 p=.005). This suggests that higher support for government intervention in healthcare was related to more deaths per total positive COVID-19 cases; the higher the proportion of deaths to the total number of COVID-19 cases, the more people support government stepping-in. Specific to high-income countries is the positive association between how much people support the government intervening in healthcare with how much the government spends in healthcare (β=.03 p=.03) and how much they have to pay out of their own pocket (β=.03 p<.0001).

Not consistent was the association between case-fatality rates and support for government intervention. Overall, there was a negative association, suggesting that that the driving factor behind support for government intervention globally is an exponential number of positive cases, which reduces the case-fatality ratio, given that the mortality rate per 100,000 people tends to be relatively stable (*30*). But in more specific models, such as isolating low-and-middle income countries (Table 3), this association became positive, differently suggesting that the higher the proportion of deaths to the total number of COVID-19 cases, the more people support government intervention in healthcare. Moreover, restricting the analysis just to high-income countries, showed no association between supporting the government to provide healthcare and the volume of positive cases and/ or deaths from COVID-19. Thus, it is unclear which consequences arise from repeated and widespread dissemination of such health information about COVID-19 statistics. Public authorities and academic institutions have gone to great lengths to keep the public continuously informed about COVID-19 related-cases and deaths. Which exact benefits this flood of information brings is ambiguous; it has been shown to have no association with preventive behaviors and compliance with containment measures (*20*), and we show here that it predicted mixed effects on attitudes about government and healthcare.

# Data Description

**
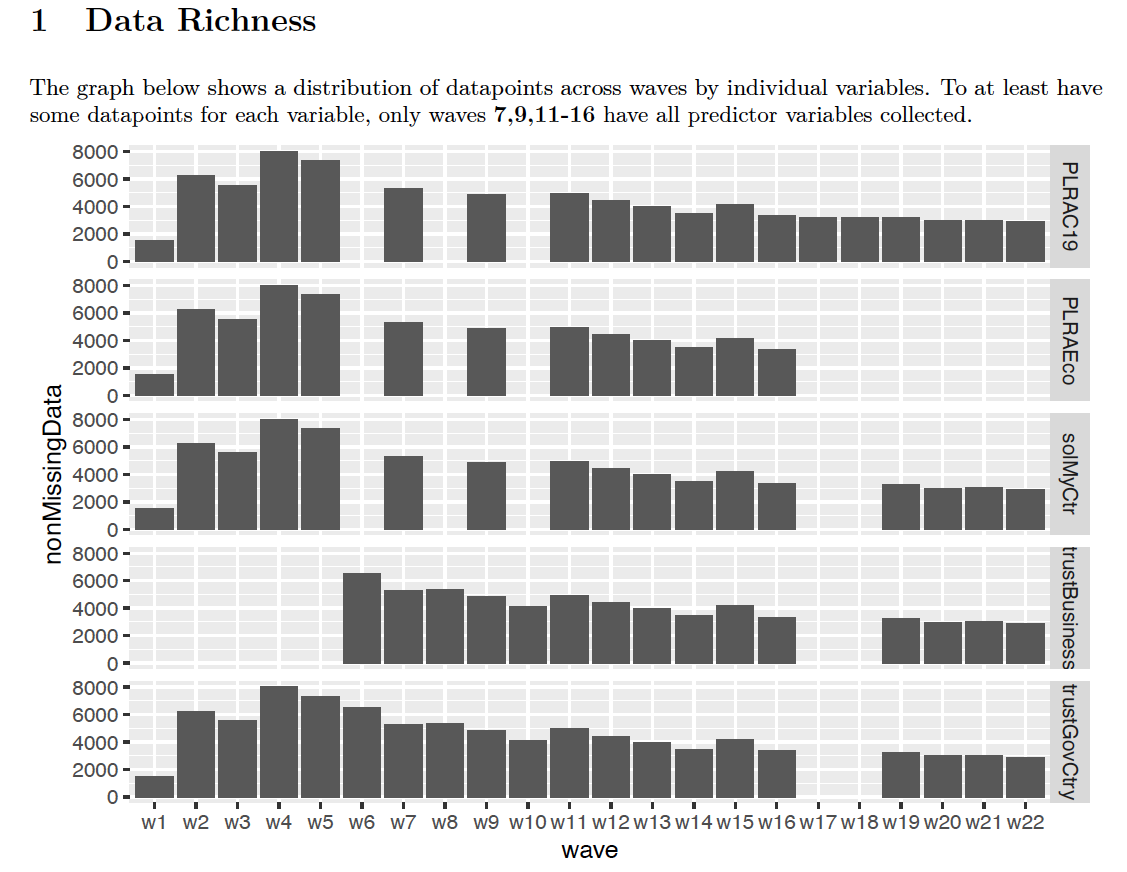
**

**
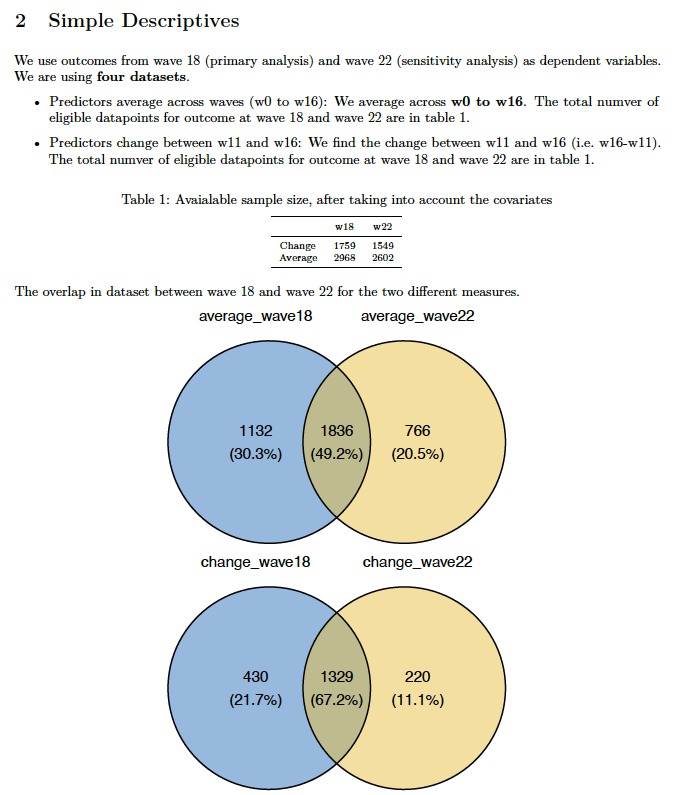
**

**
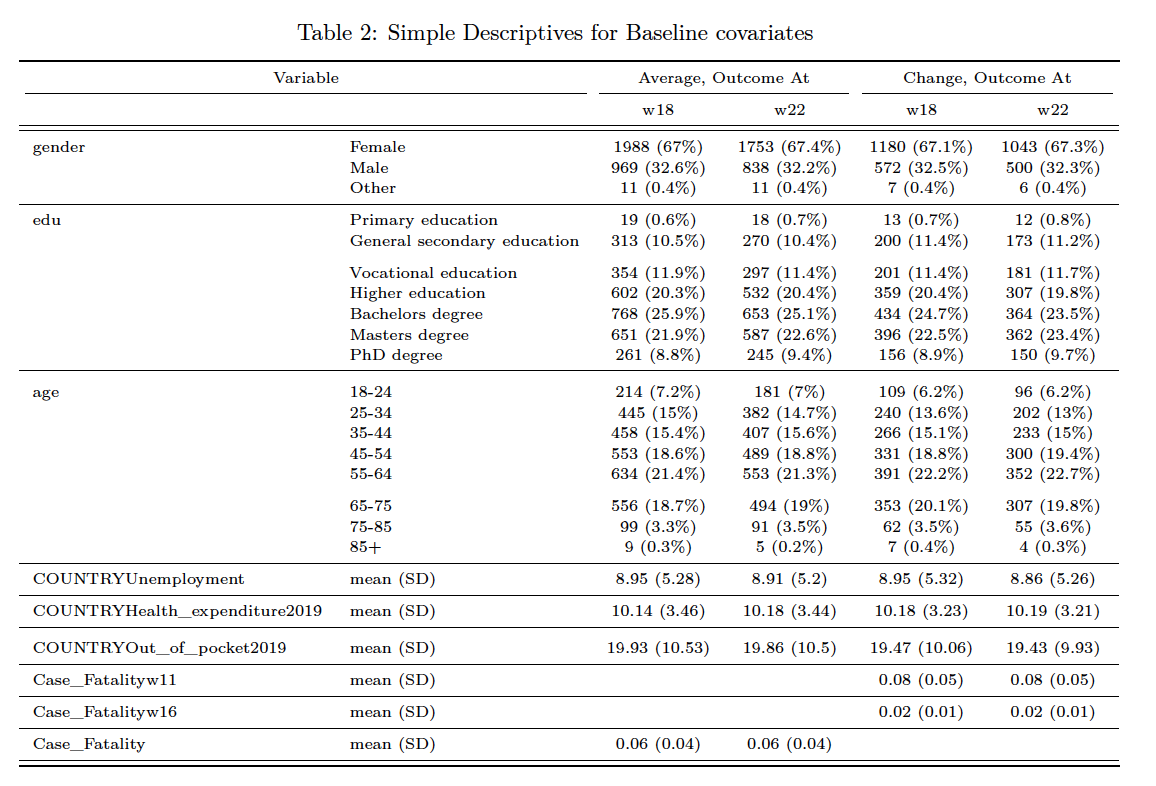
**

**
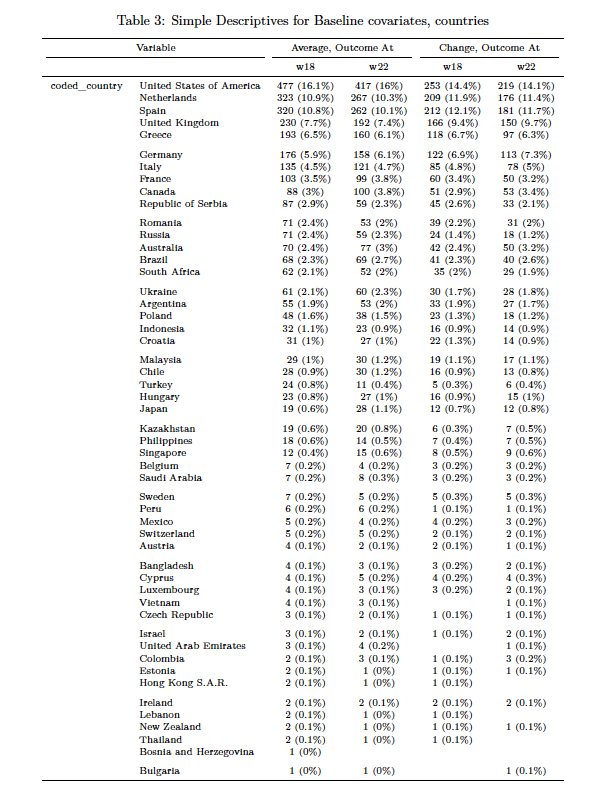
**

**
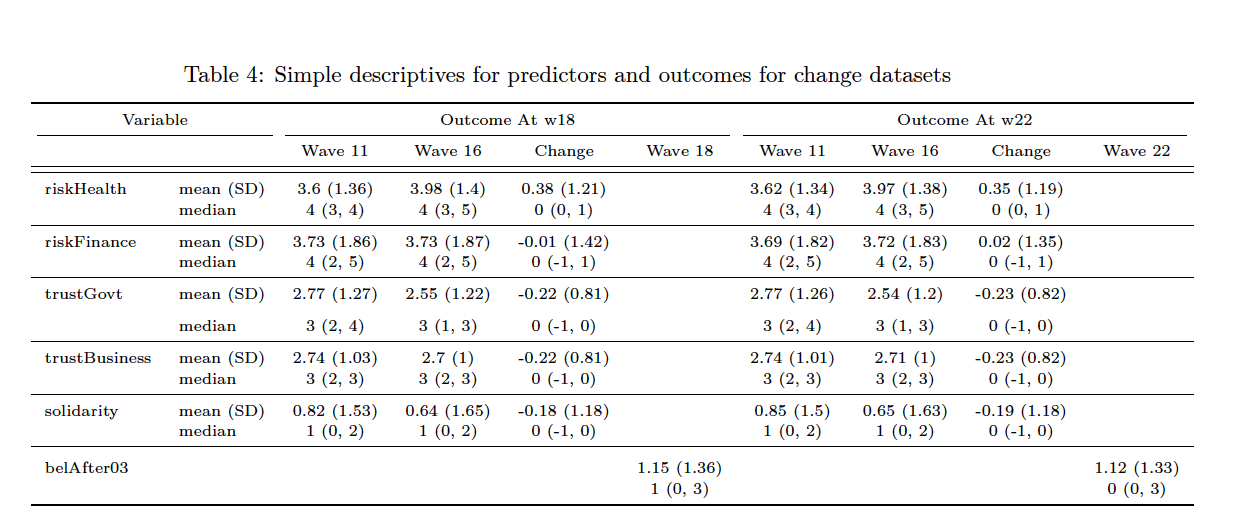
**

**
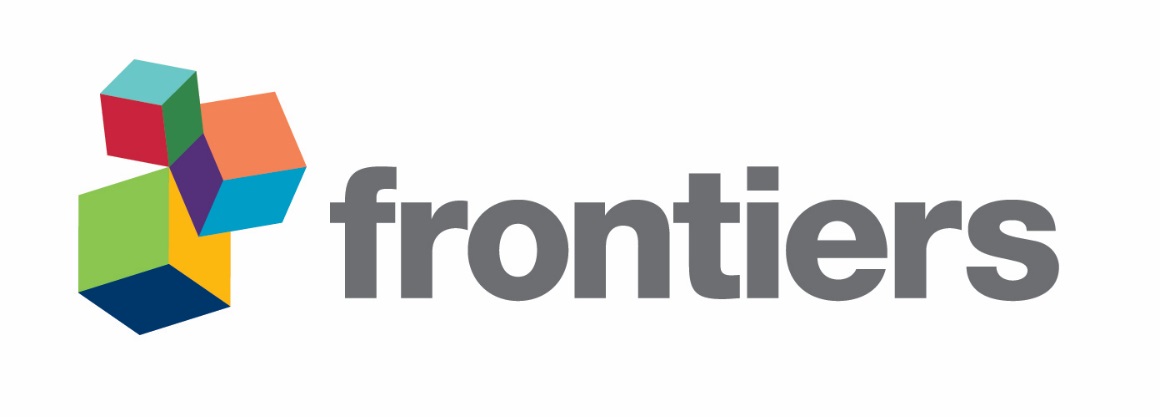
**
